# Television viewing and its association with overweight in Colombian children: results from the 2005 National Nutrition Survey: A cross sectional study

**DOI:** 10.1186/1479-5868-4-41

**Published:** 2007-09-19

**Authors:** Luis F Gomez, Diana C Parra, Felipe Lobelo, Belen Samper, José Moreno, Enrique Jacoby, Diego I Lucumi, Sandra Matsudo, Catalina Borda

**Affiliations:** 1 Health Division, Fundación FES Social, Bogota, Colombia; 2 Department of Exercise Science, Arnold School of Public Health, University of South Carolina, Columbia, SC, USA; 3 Department of Evaluation, PROFAMILIA, Bogota, Colombia; 4 Non Communicable Diseases Unit, Pan American Health Organization, Washington, D.C., USA; 5 Escuela de Medicina. Universidad Pedagógica y Tecnológica de Colombia, Tunja, Colombia; 6 Department of Evaluation, Centro de Estudos do Laboratório de Aptidão Física de São Caetano do Sul, São Caetano do Sul, Brazil; 7 Research Division, Instituto Colombiano de Bienestar Familiar, Bogota, Colombia

## Abstract

**Background:**

There has been an ongoing discussion about the relationship between time spent watching television and childhood obesity. This debate has special relevance in the Latin American region were the globalization process has increased the availability of screen-based entertainment at home. The aim of this study is to examine the association between television viewing and weight status in Colombian children.

**Methods:**

This cross sectional investigation included children aged 5 to12 yrs from the National Nutrition Survey in Colombia (ENSIN 2005). Weight and height were measured in 11,137 children in order to calculate body mass index. Overweight was defined by international standards. Time spent viewing television was determined for these children through parental reports. Multiple logistic regression analyses were conducted for different subgroups and adjusted for potential confounders in order to study the association between television viewing and weight status in this population.

**Results:**

Among the surveyed children, 41.5% viewed television less than two hours/day; 36.8% between two and 3.9 hours/day and 21.7% four or more hours/day. The prevalence of overweight (obesity inclusive) in this population was 11.1%. Children who were classified as excessive television viewers (between two and 3.9 hours/day or 4 or more hours/day) were more likely to be overweight (OR: 1.44 95% CI: 1.41–1.47 and OR: 1.32 95% CI: 1.30–1.34, respectively) than children who reported to watch television less than 2 hours/day. Stratified analyses by age, gender and urbanization levels showed similar results.

**Conclusion:**

Television viewing was positively associated with the presence of overweight in Colombian children. A positive association between urbanization level and television viewing was detected. Considering that the majority of Colombian children lives in densely populated cities and appear to engage in excessive television viewing these findings are of public health relevance for the prevention of childhood obesity.

## Background

Childhood obesity is associated with the presence of cardiovascular disease risk factors such as hyperlipidemia, hypertension, glucose intolerance, as well as their clustering [1]. Moreover, overweight children and adolescents are at increased risk for obesity and related cardio-metabolic consequences during adulthood, thus entailing that obesity prevention should begin during childhood [2,3].

The prevalence of childhood obesity has reached epidemic proportions in developed nations [4]. Recent data from the United States indicates that 37.2% of children 6 to 11 years old are overweight [5]. In developing countries such as those located in the Latin American region, childhood obesity is also emerging as a public health threat [6,7]. For example, in 1998 the prevalence of overweight among 5–11 y old Mexican children reached 19.5% [8]. Nevertheless, in a region where childhood malnutrition continues to be a top public health priority, data regarding the prevalence and trends of childhood obesity are scarce [9]. In addition, information about correlates of childhood obesity such as excessive TV viewing or decreased physical activity levels is limited.

Rapid processes of industrialization coupled with accelerated epidemiologic and nutritional transitions have been linked to the rising prevalence of childhood obesity in developing countries [10-15]. In this context, increase in time devoted to sedentary activities and reduced levels of physical activity, in conjunction with energy-dense diets, are considered factors contributing to its development [16]. In particular, television (TV) viewing has been extensively studied in relation to fatness, mainly among children and adolescents from developed countries [17,18]. The available evidence indicates that there is a significant association between TV viewing and obesity among children [18-21].

Despite the fact that increases in the prevalence of childhood obesity have been documented worldwide and that time devoted to TV viewing has been implicated in these trends, there is little information regarding the association between TV viewing and obesity in Latin America children [22]. This information has special relevance in a developing country like Colombia where the globalization process has increased the availability of television, computers and video games at home.

Consequently, the aim of this study is to examine the association between TV viewing and overweight (obesity inclusive) in a representative sample of 5–12 y old Colombian children living in both rural and urban areas.

## Methods

### Study design

The present investigation used data from the National Nutrition Survey in Colombia (in Spanish: *Encuesta Nacional de la Situación Nutricional en Colombia, ENSIN 2005*), which was conducted by the Family Welfare Colombian Institute (in Spanish: Instituto Colombiano de Bienestar Familiar, ICBF) with the logistic support of the Association for the Colombian Family Wellness (in Spanish: *Asociación Probienestar de la Familia Colombiana*, PROFAMILIA) [23]. A stratified, probabilistic, multi-stage, cluster sampling of households was performed to obtain national and sub-regional representativeness (16 sub-regions), with oversampling of rural areas and low socio-economic status (SES) groups. Logistic aspects and technical details of ENSIN can be found elsewhere [23]. The main objective of this national survey was to estimate the prevalence of nutritional problems and selected health conditions in the Colombian population. For the present study, data from 11,137 children between 5 and 12 years old (37% of the ENSIN sample) with complete information about television viewing, weight status and covariates was used. Complete information was available for 80.2% of the surveyed children. Using a propensity score we identified that those children with incomplete information had significant differences in the distribution of socioeconomic status and television viewing, however these were of low magnitude and the investigators judged that the study's internal validity will not be affected.

Seventy nutritionists received a standardized training prior to data collection with 62 of the 70 being selected as interviewers. Thirteen nutritionists were also specifically trained to take anthropometric measures.

### Measurement of the dependent variable

Weight and height were taken directly at the children's homes using standardized measuring equipment and with children in light clothing and shoes removed. Body weight was measured to the nearest 0.1 kg using a calibrated digital scale (SECA model 770) which was properly adjusted for the Colombian geographical latitude. Height was measured to the nearest 1.0 cm using a portable stadiometer (Shorr Productions). BMI was computed by dividing body weight by height squared (kg/m^2^). Overweight (obesity inclusive) was defined using the gender and age-specific cut-off points for BMI adopted by the International Obesity Task Force [24]. These cut-offs were derived by averaging the percentiles of six countries (including Brazil representing the Latin-American population), equivalent to a BMI of 25 kg/m^2 ^at age 18. This classification was selected for the present study because it constitutes the international standard to define childhood overweight, enabling cross-country comparisons.

### Measurement of TV viewing

TV viewing and video game use were determined by asking the following question: "*During the last seven days, did __________ (child's name) watch television or play video games?*" Informers who provided a positive answer were also asked about the frequency of the event: "*How many days?*" followed by "*How much time did _________ (child's name) usually spend during one of those days watching television or playing video games?*". If the interviewed parent or guardian could not answer the last question due to the variability of the report from day to day, the interviewer would then ask: "*What is the total amount of time that __________ (child's name) spent over the last seven days watching television or playing video games?*".

Based on the combined TV viewing and video-game use information, children were then classified into three groups: less than two hours per day, 2 to 3.9 hours per day, and four or more hours per day. Current public health and clinical recommendations regarding TV viewing in the pediatric setting informed the selection of this threshold (≥ 2 hours/day) to study its effects on children's weight status [25,26].

For the purposes of this study, we only refer to TV viewing since the majority of the children belonged to low SES groups and did not have access to video-games.

### Measurement of covariates

The following covariates were included, age (stratified in two groups 5 to 8 years and 9 to 12 years), gender, urbanization levels, and socioeconomic strata (SES) of the family. Based on their population density, cities and towns included in the study were divided into four urbanization levels. Urbanization level I included mostly rural settlements with 10,000 inhabitants or less. Urbanization level II included cities or towns with 10,001 to 30,000 inhabitants. Urbanization level III were cities with 30,001 to 100,000 inhabitants and urbanization level IV were cities with more than 100,000 inhabitants.

The SES of the child's family was determined according to the national SISBEN index which takes into account socio-demographic characteristics (family composition, employment status, family income, and education level), living conditions (construction type and materials), and access to public utilities (sewer, electricity, potable water and garbage collection) [27]. Based on this information, six levels are defined with one being the poorest and six being the wealthiest. For this study, levels 3 to 6 were collapsed into one group to improve the efficiency of the analyses.

### Statistical analysis

The distribution of selected covariates by TV viewing group was evaluated using Pearsons' Chi square test. The association between TV viewing and overweight (obesity inclusive) was assessed using multiple logistic regression analysis while adjusting for potential confounders (gender, age groups, SES and urbanization levels). In addition, stratified analyses were performed by age, gender, urbanization level, and SES of the family. Collinearity was examined using regression diagnostic tests including variance inflation factor (VIF) with their values always less than 0.99 [28]. The analysis took into account an unequal selection of probabilities resulting from the complex sampling design. All the statistical analyses were performed using STATA while considering sampling strata, sample units and weights [29].

## Results

### Study population characteristics

Gender distribution for the study sample (n = 11,137) was approximately equal (49.9% males) and the mean age was 8.5 y (SD = 2.28) with 50.3% of the children being in the 9–12 y old range. Approximately 41.1% of the children were in the lowest SES. Most of the surveyed children (71.3%). lived in rural areas and urban settlements with populations of less than 10.001 inhabitants

### Prevalence of TV viewing

For the study population 41.5% of parents reported their child's TV viewing level to be < 2 hours/day, 36.8% between 2 and 3.9 hours/day, and 21.7% to be 4 or more hours/day. Excessive TV viewing (≥ 2 hours/day) was more prevalent among older children (62.6%; p < 0.001), males (60.6%; p < 0.001), those with middle to high SES (levels 3 – 6; 66.5%; p < 0.001), and in children living in the most urbanized areas (level IV; 71.3 %; p < 0.001) (Table 1).

**Table 1:** Prevalence of television viewing by sociodemographic characteristics among 11,137 children aged 5 to 12 years. Analysis conducted from the National Nutrition Survey (ENSIN) Colombia. 2005

**Characteristics**	n*	**< 2 hrs/day**		**2 – 3.9 hrs/day**		**≥ 4 hrs/day**		*p*
			
		P**	SE	P**	SE	P**	SE	
**All participants**	11,137	41.5	0.03	36.8	0.02	21.7	0.02	
**Age groups, yrs**								
5–8	5,539	45.7	0.04	34.8	0.02	19.5	0.02	< 0.001
9–12	5,598	37.4	0.03	38.7	0.03	23.9	0.02	
**Sex**								
Male	5,568	39.4	0.05	38.1	0.03	22.5	0.03	< 0.001
Female	5,569	43.6	0.03	35.4	0.02	21.0	0.02	
**Socio Economic Status**								
Level 1 (Lowest)	4,576	56.0	0.04	28.9	0.03	15.1	0.02	< 0.001
Level 2	4,124	34.7	0.04	41.1	0.03	24.2	0.03	
Level 3 to 6 (Middle – high)	2,437	33.5	0.05	40.3	0.04	26.2	0.03	
**Levels of urbanization**								
Level I (rural and urban areas with 10.00 inhs or less)	7,946	51.1	0.04	32.9	0.03	16.0	0.03	< 0.001
Level II (10.001 to 30.000 inhs)	833	30.4	0.01	42.1	0.01	27.5	0.01	
Level III (30.001 to 100.000 inhs)	1,292	33.7	0.08	39.4	0.07	26.9	0.03	
Level IV (more than 100.000 inhs)	1,066	28.7	0.01	42.1	0.01	29.2	0.01	

*Unweighted sample size.

**Weighted percentage (P) and standard error (SE)

### Prevalence of overweight

Of the studied children, 11.1% were classified as overweight (obesity inclusive) with a higher prevalence among younger children (5–8 y: 11.3%; p < 0.001), those living in the most urbanized areas (level III and IV: 14.1% and 14.6% respectively; p < 0.001), and those in middle to high SES level (levels 3–6: 17.2 %; p < 0.001) (Table 2).

**Table 2:** Prevalence of overweight (obesity inclusive) by selected sociodemographic characteristics and television viewing among 11,137 children aged 5 to 12 years. Analysis conducted from the National Nutrition Survey (ENSIN) Colombia. 2005

**Characteristics**	P*	**SE**	***p***
**All participants**	11.1	0.05	
**Age groups, yrs**			
5–8	11.3	0.08	< 0.001
9–12	10.9	0.06	
**Sex**			
Male	10.0	0.08	< 0.001
Female	12.2	0.04	
**Socio Economic Status**			
Level 1 (Lowest)	5.0	0.08	< 0.001
Level 2	11.8	0.06	
Levels 3 to 6 (Middle-high)	17.2	0.05	
**Levels of urbanization**			
Level I (rural an urban areas with 10.000 inhs or less)	8.5	0.07	< 0.001
Level II (10.001 to 30.000 inhs)	11.6	0.03	
Level III (30.001 to 100.000 inhs)	14.1	0.09	
Level IV (more than 100.000 inhs)	14.6	0.01	
**Television viewing exposure levels**			
< 2 hrs/day	8.5	0.05	< 0.001
2 – 3.9 hrs/day	13.5	0.10	
4 or more hrs/day	13.0	0.05	

*Weighted percentage (P) and standard error (SE)

### Associations between TV viewing and overweight

In the logistic regression models, children classified as excessive TV viewers (2 to 3.9 hours/day or 4 or more hours/day) were more likely to be overweight (OR: 1.44 95% CI: 1.41–1.47 and OR: 1.32 95% CI: 1.30–1.34, respectively) than those who watched less than 2 hours/day. This pattern of association was also found in analysis stratified by gender, age and urbanization levels. (Table 3)

**Table 3:** Odds Ratios for overweight (obesity inclusive) by sex, age groups, and urbanization levels among 11,137 children aged 5 to 12 years, Analysis conducted from the National Nutrition Survey (ENSIN) Colombia. 2005

**Television viewing exposure levels**		**Adjusted OR**	**95% CI**	***p***
**a**	**Total population**			
	< 2 hrs/day	1.00	Referent	
	2 – 3.9 hrs/day	1.44	(1.41–1.47)	< 0.001
	≥ 4 hrs/day	1.32	(1.30–1.34)	< 0.001
**b**	**All males**			
	< 2 hrs/day	1.00	Referent	
	2 -3.9 hrs/day	1.51	(1.46–1.56)	< 0.001
	≥4 hrs/day	1.38	(1.34–1.42)	< 0.001
**b**	**All females**			
	< 2 hrs/day	1.00	Referent	
	2 -3.9 hrs/day	1.39	(1.34–1.44)	< 0.001
	≥ 4 hrs/day	1.29	(1.26–1.32)	< 0.001
**c**	**All children aged 5 to 8 ys**			
	< 2 hrs/day	1.00	Referent	
	2 -3.9 hrs/day	1.19	(1.15–1.22)	< 0.001
	≥ 4 hrs/day	1.32	(1.29–1.36)	< 0.001
**c**	**All children aged 9 to 12 ys**			
	< 2 hrs/day	1.00	Referent	
	2 -3.9 hrs/day	1.78	(1.73–1.84)	< 0.001
	≥ 4 hrs/day	1.40	(1.36–1.43)	< 0.001
**d**	**Level I (rural and urban areas with 10000 inhabitants or less)**			
	< 2 hrs/day	1.00	Referent	
	2 -3.9 hrs/day	1.29	(1.24–1.34)	< 0.001
	≥ 4 hrs/day	1.49	(1.46–1.52)	< 0.001
**d**	**Level II to IV (10001 inhabitants and more)**			
	< 2 hrs/day	1.00	Referent	
	2 -3.9 hrs/day	1.56	(1.53–1.60)	< 0.001
	≥ 4 hrs/day	1.26	(1.24–1.29)	< 0.001

a Adjusted by sex, age groups, SES and urbanization levels.

b Adjusted by age groups, SES and urbanization levels.

c Adjusted by sex, SES and urbanization levels.

d Adjusted by sex, age groups and SES.

In addition, the strength of association between TV viewing and weight status was greater for those in the 2 – 3.9 hours/day group than for those in the 4 or more hours/day group, with the exception of the 5 to 8 y old group and those who lived in rural areas and urban settlements of less than 10,001 inhabitants.

## Discussion

In this study we found a positive association between excessive TV viewing and the presence of overweight among Colombian children. This association was observed for children of different gender, age groups (5–8 and 9–12 y old) and for children living in different urbanization levels.

The magnitude of the associations detected between TV viewing groups and weight status ranged from (OR) 1.32 to 1.44 for the total sample and from 1.19 to 1.78 in the sub-group analyses. Previous studies exploring the association between electronic media use and health-related outcomes have found conflicting results. Some investigations have not detected significant associations [30,31] but most have reported a positive relationship [17,32-36]. The association between TV viewing and childhood obesity has been found consistently across different sociodemographic groups, as explored by studies conducted among different age groups, [32,33,35,37] and in children from developed[17] and developing countries [22]. Furthermore, a recent meta-analysis found a positive, yet weak relationship, between fatness and TV viewing [18]. The findings of the present study are in concordance with previous evidence on the topic and have important public health implications [38].

Among children aged 5–8 yrs and children living in rural or urban areas with less than 10,001 inhabitants, we found increasing odds for overweight as TV viewing increased. The stability of the estimates we obtained for the association between weight status and TV viewing may have been affected by information biases related to this last variable. For example, a recent study indicates that having a TV in the children's bedroom should become an important control variable in studies where estimates are obtained by proxy report [39]. This is because parents overestimate children's TV viewing time when no TV is present in the bedroom but underestimate it when there is a TV set in the bedroom [39]. In this study we did not have this information to adjust for, and determine the direction and magnitude of the error in the exposure variable.

Parental reports of TV viewing time in older children (9–12 y old) might not be as adequate because these children are more independent than their younger counterparts and their parents could have less control over their daily activities, thus overestimating their TV viewing time.

Similarly, the quality of information regarding TV viewing time might be better for children of low SES living in rural or small urban settlements where, in most of the cases, there is only one TV. In such cases parents will probably have a better idea of their children's TV viewing time. More detailed information on the children's TV viewing patterns will be an important addition to future studies exploring health-related effects of TV viewing and other electronic entertainment. While the effect of excessive TV viewing time on adiposity could be explained by the displacement of physically active pursuits and/or increases in energy intake while viewing TV, [40] an important question is how these factors behave differently depending on the cultural and social contexts. Social and physical environments have been recognized as significant determinants of health behaviors [40,41]. Aspects such as living in a safe place that supports physical activity play an important role in the prevention of childhood obesity [41]. It may be the case that families living in low SES areas with high crime rates, choose to restrict children's outdoor play and replace this time with indoor activities such as TV viewing [42]. Likewise, food advertising and the availability of fast food products are important factors to consider in the development of childhood obesity in the Latin American region [43]. The availability of energy-dense foods in developing countries has certainly increased as a result of the industrialization and globalization processes [44]. Further studies will need to explore the extent to which these products are being advertised to children and youth and whether an association exists between food advertisement and childhood obesity in Latin America. All of these aspects should be contemplated in future studies in order to better understand the myriad of interacting factors driving the relationship between TV viewing and childhood obesity in particular for Latin American youth.

The findings of this study point to the existence of a positive association between the level of urbanization with both TV viewing time and prevalence of childhood obesity. The fact that the most urbanized areas have more access to television as an indirect result of the globalization process might explain these results. The Latin American region is urbanizing at an extremely rapid pace. Currently, 75% of the population lives in urbanized areas and it is predicted that this proportion will increase to 84% by 2030 [45]. As most Latin American countries experience rapid epidemiological and nutritional transitions it is important to recognize that increases in the prevalence of childhood obesity occur as a function of these epidemiological and societal trends of development [46]. Therefore, children living in urban areas and exposed to excessive TV viewing should be considered a group at particular risk for childhood obesity.

Several strengths can be identified in this study. To our knowledge, this is the first investigation to explore the association between TV viewing and overweight in a national representative sample of children from a Latin American country. The use of data from a national survey allowed us to contrast the association between TV viewing and weight status by children living in different urbanization levels and SES. The study previously conducted in Mexico was restricted to children who resided in the Federal District [22]. On the other hand, some limitations need to be discussed. The methodology used for the quantification of TV viewing might be affected by information biases related to inaccuracies in the parental reports of TV watching. Distortions such as lack of awareness of their child's TV viewing habits, recall bias, desire to please, and reluctance to tell the truth might be present. Another aspect to be considered is the difficulty to administer surveys in low educated populations from developing countries. Nevertheless, proxy reports are the most used method (and possibly the only feasible one) to measure TV viewing time in a population survey of children between 5 and 12 y old [47]. These issues emphasize the need of improving the design of instruments to better measure TV viewing time in ways that meet the specific needs of Latin America populations. In addition, the cross-sectional nature of this study does not allow to establish a causal relationship between TV viewing and children's weight status. Despite these limitations, the results of this study should promote interventions to limit TV viewing as part of childhood obesity prevention programs in the region.

## Conclusion

Time spent in sedentary activities in particular TV viewing is emerging as a risk factor for different health outcomes in children including obesity and related cardio-metabolic disarrays. There is a growing concern that rapid processes of globalization and urbanization occurring in developing regions like Latin America will impact the prevalence of childhood obesity in part as a result of increased availability of television at home. Our findings suggest that TV viewing is positively associated with the presence of overweight and obesity among Colombian children. At the same time, both TV viewing and the prevalence of overweight were found to be positively associated with urbanization level. The public health implications of these findings are considerable, since the majority of Colombian children live in densely populated areas and engage in excessive TV viewing. These trends are a call for action for the development and implementation of programs and national policies for the prevention of childhood obesity in Colombia and the Latin American region. An important component of such programs should be strategies to limit TV viewing among children and adolescents

## Competing interests

The author(s) declare that they have no competing interests.

## Authors' contributions

LFG conceived the study and participated in the analysis and drafting of the manuscript. DP and FL participated in the analyses and interpretation of data and in the drafting of the manuscript. BS, JM and DL provided valuable comments in the analyses and discussion sections of the manuscript. EJ, SM, CB contributed in data collection protocol for ENSIN and provided valuable comments in the drafting of the manuscript. All authors read and approved the final manuscript
